# Supplementary material for: Plasma membrane association and resistosome formation of plant helper immune receptors
Source: Proc Natl Acad Sci U S A. 2023 Jul 31;120(32):e2222036120. doi: 10.1073/pnas.2222036120 (PMC10410763; doi:10.1073/pnas.2222036120)
Supplement: Supplementary file 1 — Appendix 01 (PDF) [file pnas.2222036120.sapp.pdf]

Supplementary figures

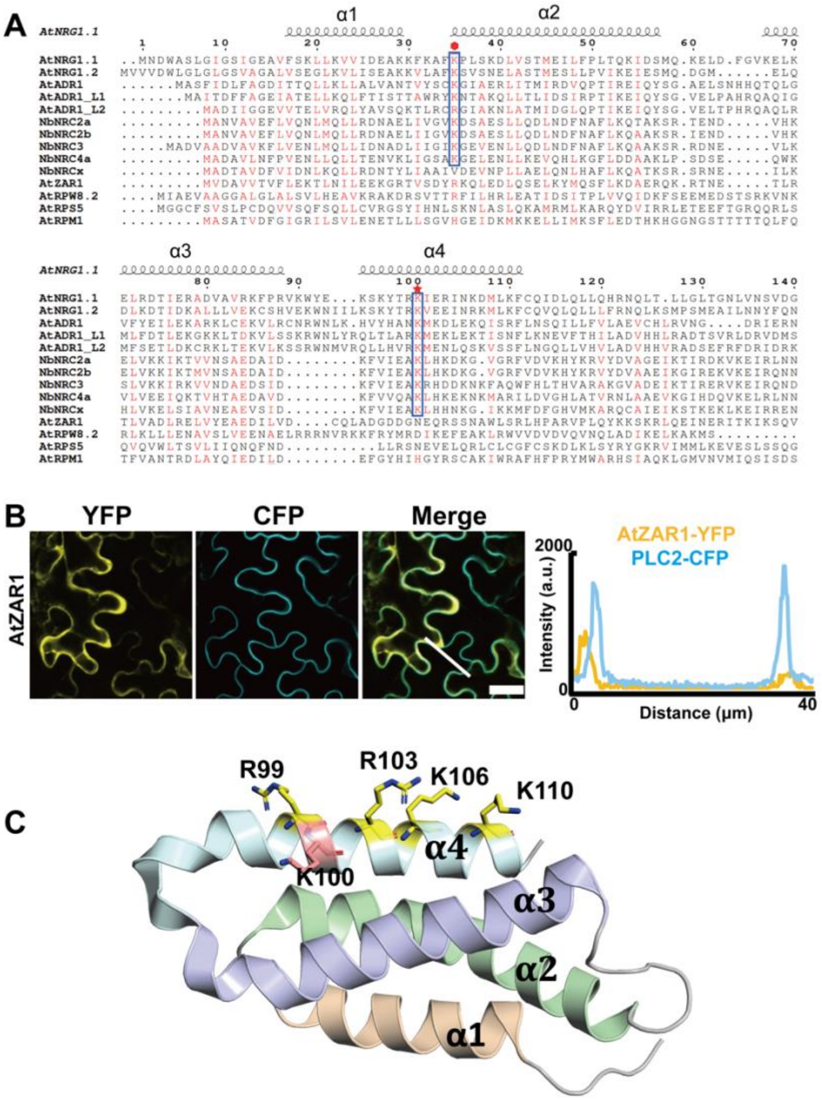

Figure S1 Identification of conserved positively charged residues in plant helper NLRs. **(A)** Sequence alignment in the CC^R and CC domains of helper NLRs and some sensor CNLs from Arabidopsis and *Nb*. The alignment was performed with ClustalX and the figure was prepared using ESPript. Red asterisk points to the position of AtNRG1.1 K100, and red hexagon points to the position of AtADR1_L1 K30. **(B)** Confocal assay showing that resting state AtZAR1 mainly localizes in the cytosol. Fluorescence intensities were measured along the white line depicted in the merge images. Bars, 25 μm. **(C)** Cartoon presentation of AtNRG1.1 CC^R structure (PDB:7L7W) with R99 K110, R103, K106 and K110 shown as sticks.

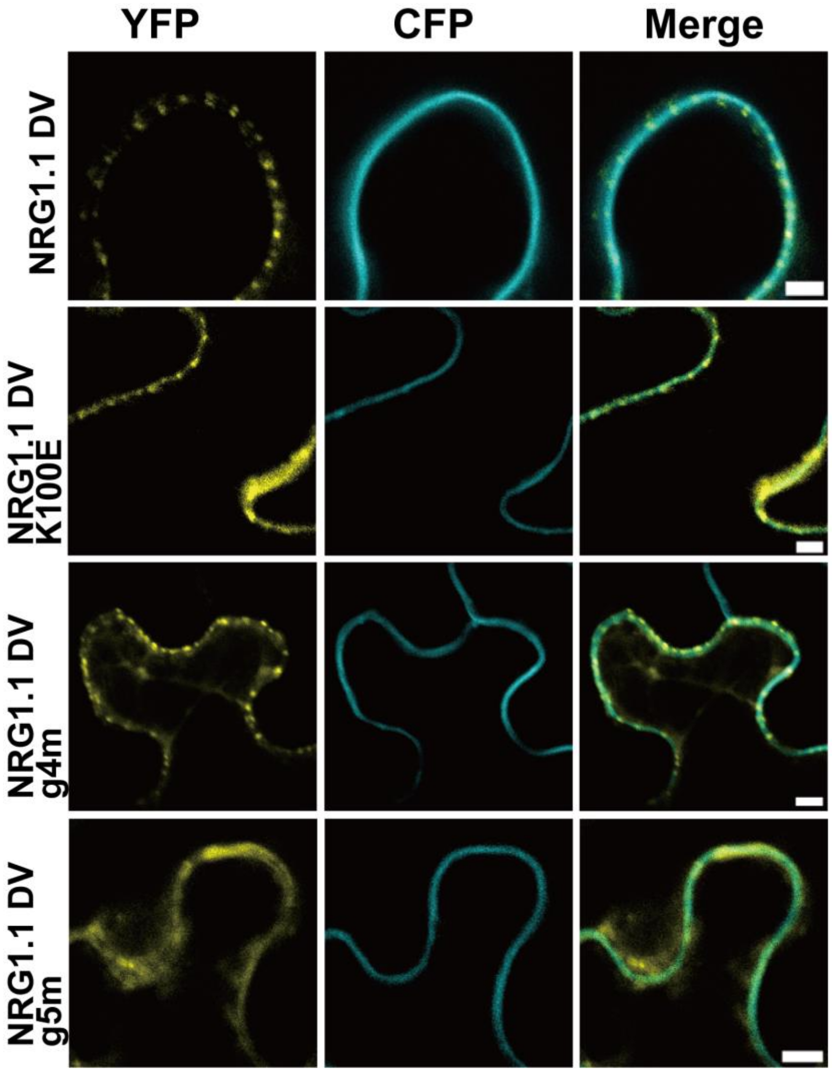

Figure S2 Peripheral images of the confocal assays in Figure 2C.

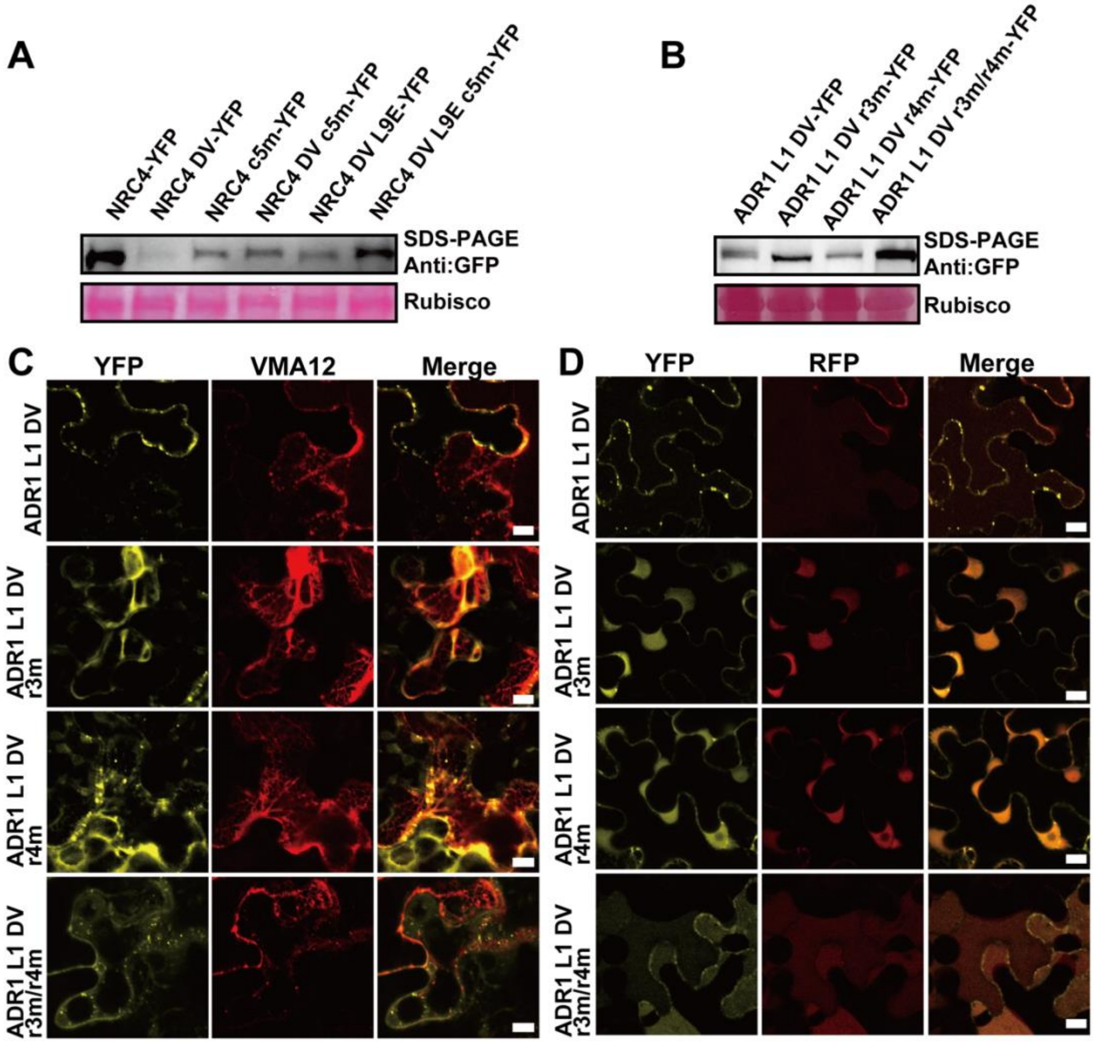

Figure S3 (A) Protein expression levels of NbNRC4 and NbNRC4 DV relevant mutants in Figure 3B. **(B)** Protein expression levels of AtADR1_L1 relevant mutants assayed by immunoblot. Confocal microscopy assays showing if the mis-localized ADR1_L1 DV mutants co-localize with the ER marker VMA12 (**C**) or cytosolic RFP (**D**). The indicated proteins fused with a C-terminal YFP were transiently co-expressed with the ER marker VMA12 fused to RFP or RFP in *Nb* leaves and confocal images were taken at 32-36h post infiltration. Confocal images are single plane secant views. Images are single plane secant views. Merge means merged images between YFP and RFP images.

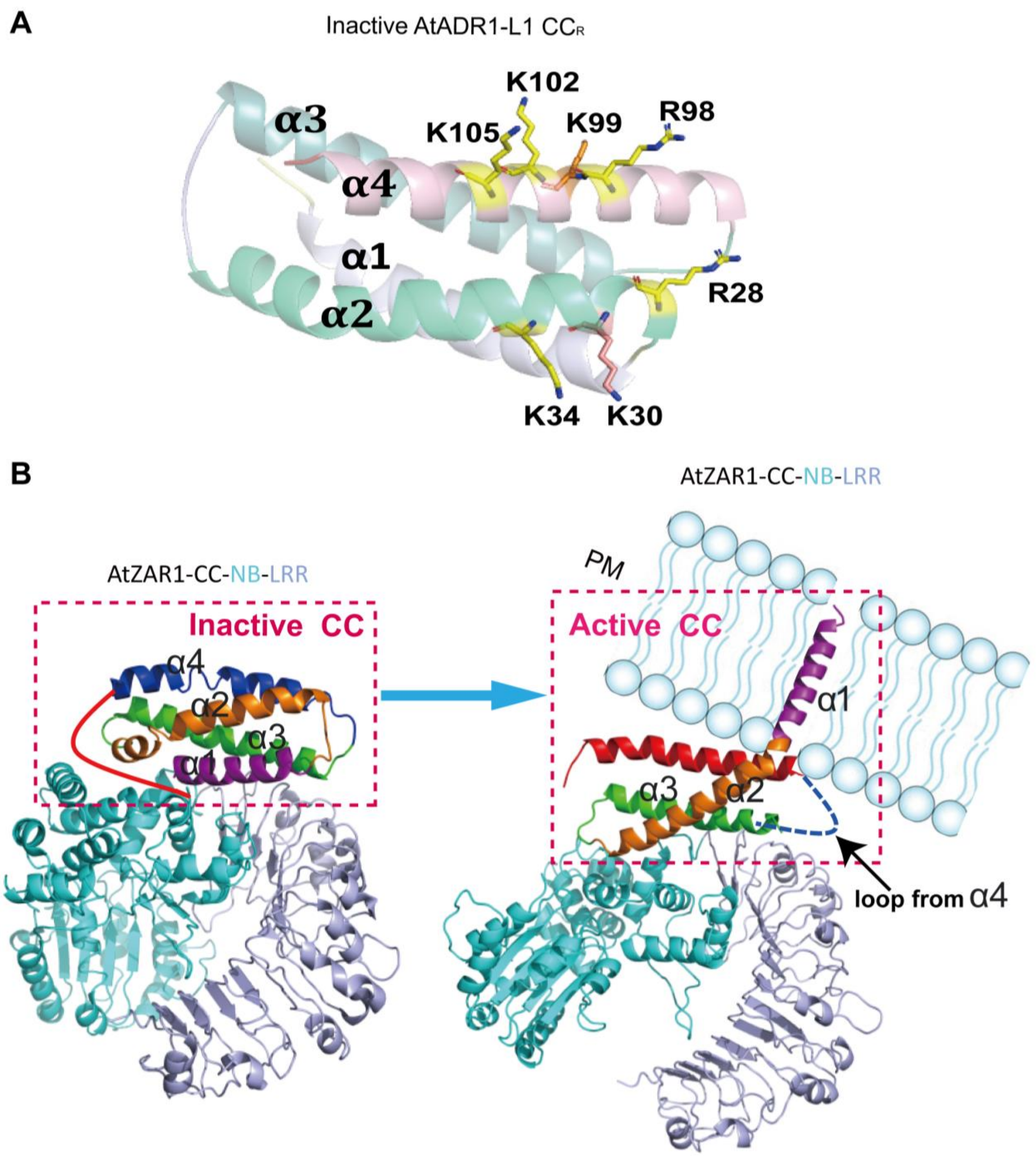

Figure S4 (A) Cartoon presentation of an AlphaFold AtADR1-L1 structure highlighting the positively charged residues involved in r3m and r4m. **(B)** Cartoon presentation of AtZAR1 in resting state (PDB: 6J5W) and in active state (PDB: 6J5T) highlighting the conformational changes in the CC domain and PM association.

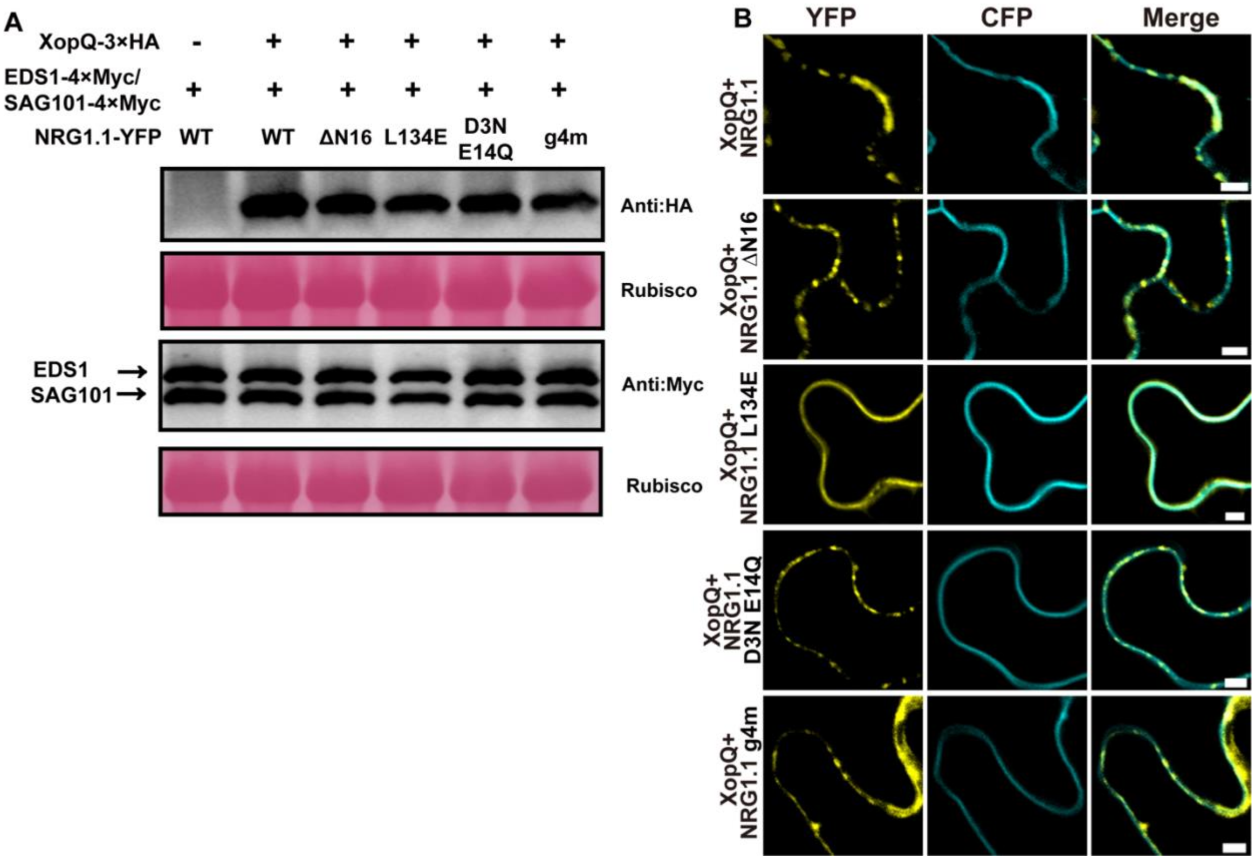

Figure S5 (A) Protein levels of XopQ, EDS1 and SAG101 from experiments displayed in Figure 5A and 4B. HA-tagged XopQ and Myc-tagged EDS1/SAG101 were separated by SDS-PAGE and blotted for anti-HA and anti-Myc, respectively. **(B)** Peripheral images of the confocal assays in Figure 5D-5H.

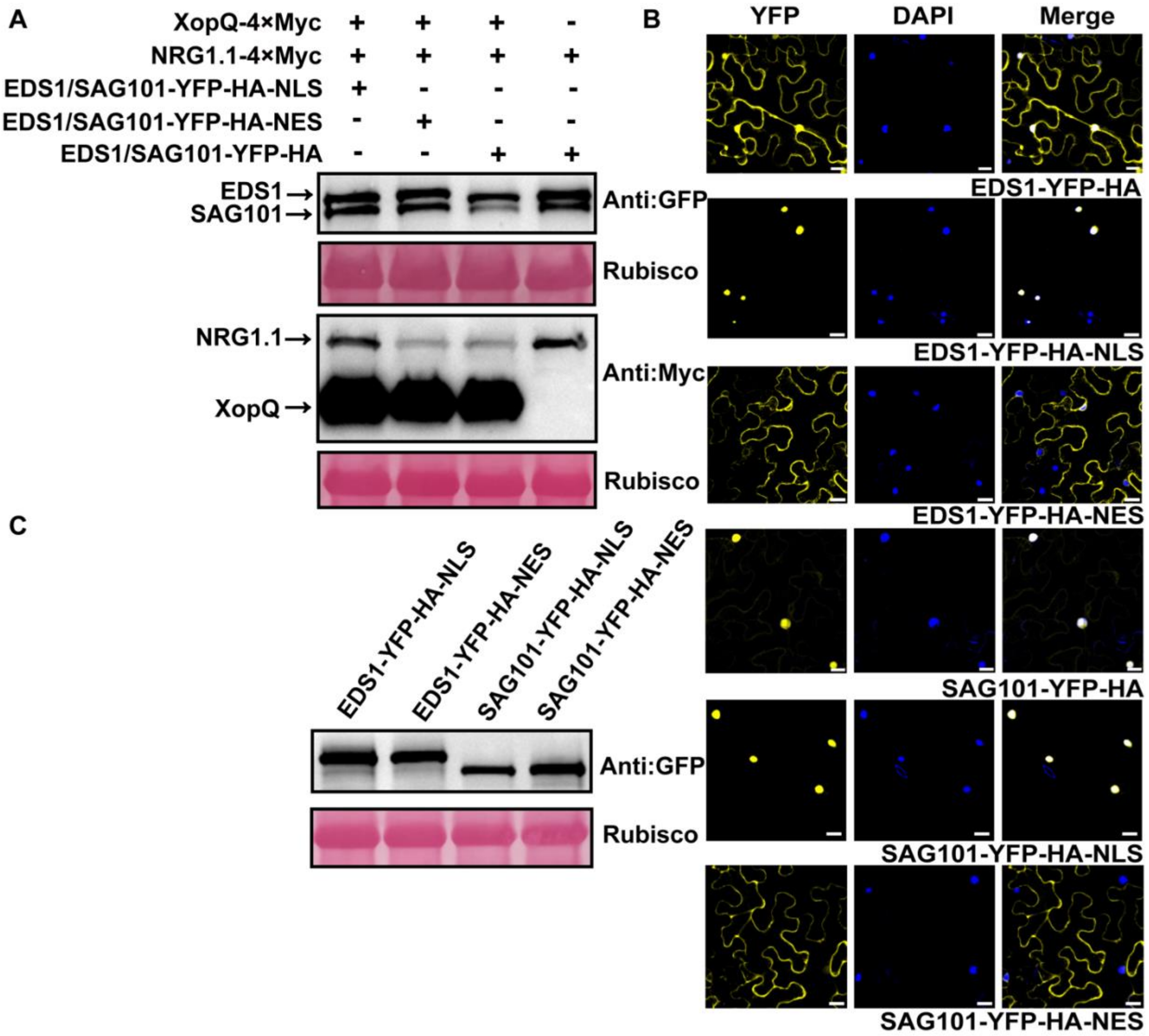

Figure S6 (A) Protein expression levels of samples from experiments displayed in Figure 6A. Total proteins were extracted from samples harvested at 26h post infiltration. Target proteins were separated by SDS-PAGE and blotted with the appropriate antibodies. **(B)** Confocal images showing the subcellular localization of EDS1-YFP-HA, EDS1-YFP-HA-NLS, EDS1-YFP-HA-NES, SAG101-YFP-HA, SAG101-YFP-HA-NLS and SAG101-YFP-HA-NES. Images were photographed at 48h post infiltration in *Nb* leaves. Nuclei were stained with DAPI (4’, 6-diamidino-2-phenylindole). Bars, 20 μm. **(C)** Protein accumulation of samples in (B) detected with anti-GFP antibody.

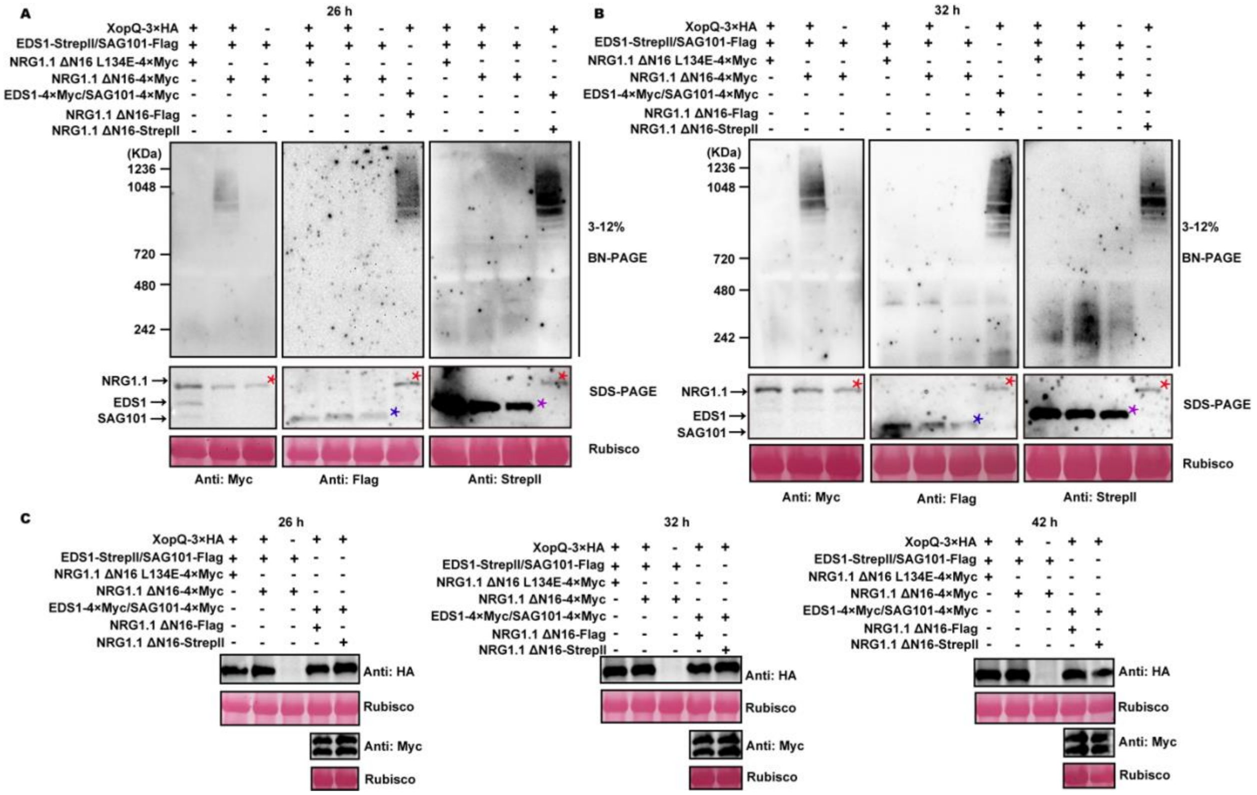

Figure S7 BN-PAGE assays showing that EDS1/SAG101 is not observed in an oligomeric complex with NRG1.1 at 26h **(A)** or 32h (**B)** after co-expression with XopQ in *Nb epss*. Red, purple and blue asterisks indicate NRG1.1, EDS1 and SAG101 bands, respectively. **(C)** Protein expression of XopQ, EDS1 and SAG101 as displayed in Figure 6C and Figure S7A and S7B was verified by SDS-PAGE. XopQ, EDS1/SAG101 were detected with anti-HA antibody and anti-Myc antibody, respectively.

Table S1 Gene ID of the 69 helper NLR homologs used in the alignment by CLustalW.

Gene	Gene ID (databases)[1]	Plant species
AtNRG1.1	At5g66900.1 (TAIR)	*Arabidopsis thaliana*
AtNRG1.2	At5g66910.1 (TAIR)	*Arabidopsis thaliana*
NbNRG1	NbS00018358g0013.1 (SGN)	*Nicotiana benthamiana*
AlNRG1	2614272850 (IMG)	*Arabidopsis lyrata*
AhNRG1	2614358615 (IMG)	*Arabidopsis halleri*
CrNRG1	2614531086 (IMG)	*Capsella rubella*
EhNRG1	2615507434 (IMG)	*Eutrema halophilum*
CsNRG1	2615787498 (IMG)	*Citrus sinensis*
CcNRG1	2614549921 (IMG)	*Citrus clementina*
PpNRG1	2615813723 (IMG)	*Prunus persica*
VvNRG1.1	649552880 (IMG)	*Vitis vinifera*
VvNRG1.2	649554972 (IMG)	*Vitis vinifera*
SpNRG1.1	2615336907(IMG)	*Salix purpurea*
SpNRG1.2	2615348770 (IMG)	*Salix purpurea*
RcNRG1	649572805 (IMG)	*Ricinus communis*
PtNRG1	2615290452 (IMG)	*Populus trichocarpa*
SlNRG1	Solyc02g090380.4.1 (SGN)	*Nicotiana benthamiana*
AtADR1	At1g33560.1 (TAIR)	*Arabidopsis thaliana*
AtADRL1 L1	At4g33300.1 (TAIR)	*Arabidopsis thaliana*
AtADRL1 L2	At5g04720.1 (TAIR)	*Arabidopsis thaliana*
NbADR1	Niben101Scf02422g02015.1 (SGN)	*Nicotiana benthamiana*
AlADR1	2614261905 (IMG)	*Arabidopsis lyrata*
AlADR1 L1	2614291476 (IMG)	*Arabidopsis lyrata*
AlADR1 L2	2614268259 (IMG)	*Arabidopsis lyrata*
AhADR1	2614369556 (IMG)	*Arabidopsis halleri*
CrADR1	2614512948 (IMG)	*Capsella rubella*
CrADR1 L1	2614509067 (IMG)	*Capsella rubella*
CrADR1 L2	2614505477 (IMG)	*Capsella rubella*
EhADR1	2615515499 (IMG)	*Eutrema halophilum*
EhADR1 L1	2615525947 (IMG)	*Eutrema halophilum*
EhADR1 L2	2615515499 (IMG)	*Eutrema halophilum*
GrADR1	2614796124 (IMG)	*Gossypium raimondii*
PtADR1	2615278681 (IMG)	*Populus trichocarpa*
CcADR1	2614532153 (IMG)	*Citrus clementina*
CsADR1	2615787425 (IMG)	*Citrus sinensis*
VvADR1	649539454 (IMG)	*Vitis vinifera*
SpADR1	2615316196 (IMG)	*Salix purpurea*
PpADR1	2615813687 (IMG)	*Prunus persica*
RcADR1	649560471 (IMG)	*Ricinus communis*
NbNRC2a	NbS00018282g0019.1 (SGN)	*Nicotiana benthamiana*
NbNRC2b	NbS00026706g0016.1 (SGN)	*Nicotiana benthamiana*
NbNRC3	NbS00011087g0003.1 (SGN)	*Nicotiana benthamiana*
NbNRC4a	NbS00002971g0007.1 (SGN)	*Nicotiana benthamiana*
NbNRC4b	NbS00016103g0004.1 (SGN)	*Nicotiana benthamiana*
NbNRCX	NbS00030243g0001.1 (SGN)	*Nicotiana benthamiana*
SlNRC1	Solyc01g090430.3.1 (SGN)	*Solanum lycopersicum*
SlNRC2	Solyc10g047320.2.1 (SGN)	*Solanum lycopersicum*
SlNRC3	Solyc05g009630.3.1 (SGN)	*Solanum lycopersicum*

Gene	Gene ID (databases)[1]	Plant species
SlNRC4a	Solyc04g007070.3.1 (SGN)	*Solanum lycopersicum*
SlNRC4b	Solyc04g007060.3.1 (SGN)	*Solanum lycopersicum*
SlNRC4c	Solyc04g007030.3.1 (SGN)	*Solanum lycopersicum*
SlNRCX	Solyc03g005660.4.1 (SGN)	*Solanum lycopersicum*
StNRC1a	PGSC0003DMT400066999 (SGN)	*Solanum tuberosum*
StNRC1b	PGSC0003DMT400066996 (SGN)	*Solanum tuberosum*
StNRC1c	PGSC0003DMT400066993 (SGN)	*Solanum tuberosum*
StNRC2a	PGSC0003DMT400080649 (SGN)	*Solanum tuberosum*
StNRC2b	PGSC0003DMT400080647 (SGN)	*Solanum tuberosum*
StNRC4a_1	PGSC0003DMT400041024 (SGN)	*Solanum tuberosum*
StNRC4a_2	PGSC0003DMT400069010 (SGN)	*Solanum tuberosum*
StNRC4a_3	PGSC0003DMT400069014 (SGN)	*Solanum tuberosum*
StNRC4a_4	PGSC0003DMT400041023 (SGN)	*Solanum tuberosum*
StNRC4b_1	PGSC0003DMT400019304 (SGN)	*Solanum tuberosum*
StNRC4b_2	PGSC0003DMT400019303 (SGN)	*Solanum tuberosum*
StNRC4b_3	PGSC0003DMT400019302 (SGN)	*Solanum tuberosum*
StNRC4b_4	PGSC0003DMT400019327 (SGN)	*Solanum tuberosum*
SmNRC1	SMEL4.1_12g003240.1 (SGN)	*Solanum melongena*
SmNRC3	SMEL4.1_10g001370.1 (SGN)	*Solanum melongena*
SmNRC4a	SMEL4.1_11g018090.1 (SGN)	*Solanum melongena*
SmNRC4b	SMEL4.1_11g018170.1 (SGN)	*Solanum melongena*

[1]TAIR, IMG and SGN are abbreviated from Arabidopsis Information Resource, Integrated Microbial Genomes and Solanaceae Genomics Network databases.
